# Seasonal variation in the response of a monoecious crop to increased temperature and fertilizers

**DOI:** 10.3389/fpls.2022.1012859

**Published:** 2022-10-07

**Authors:** Maribel López-Atanacio, Rodrigo Lucas-García, Victor Rosas-Guerrero, Lorena Alemán-Figueroa, José Gabriel Kuk-Dzul, Giovanni Hernández-Flores

**Affiliations:** ^1^ Posgrado en Recursos Naturales y Ecología, Facultad de Ecología Marina, Universidad Autónoma de Guerrero, Acapulco, Guerrero, Mexico; ^2^ Escuela Superior en Desarrollo Sustentable, Universidad Autónoma de Guerrero, Tecpan de Galeana, Guerrero, Mexico; ^3^ Consejo Nacional de Ciencia y Tecnología (CONACYT) - Facultad de Ecología Marina, Universidad Autónoma de Guerrero, Acapulco, Guerrero, Mexico; ^4^ Consejo Nacional de Ciencia y Tecnología (CONACYT) - Escuela Superior de Ciencias de la Tierra, Universidad Autónoma de Guerrero, Taxco el Viejo, Guerrero, Mexico

**Keywords:** climate change, *Cucurbita pepo*, ecosystem service, floral traits, global warming, insect-pollinated crops, pollination, vermicompost

## Abstract

Climate warming may affect the performance of plants directly through altering vegetative or reproductive traits, and indirectly through modifying interactions with their pollinators. On the other hand, the addition of fertilizers to the soil may increase the quantity and quality of floral rewards, favoring the visitation of pollinators and, consequently, the reproductive success of plants. However, it is still unknown whether fertilizers may counteract the effects of increased temperature on the vegetative, floral, and reproductive traits of plants, as well as on the interaction with their pollinators. The aim of this study is to evaluate the effects of the input of organic and synthetic fertilizers on several vegetative and floral traits, and on the rate of legitimate floral visitors and reproductive success of the squash during two seasons, under a scenario of an increase in ambient temperature. During the dry and the rainy seasons, three vegetative, eleven floral, and two reproductive traits, as well as the duration of visits and visitation rate of legitimate floral visitors were evaluated in squash plants distributed into six treatments in a bifactorial design: temperature (ambient or elevated temperature) and fertilizer (organic, synthetic or without supplementary fertilizers). Contrary to our predictions, we found that an increase of ~1.5°C in ambient temperature, positively influenced several vegetative, floral, and reproductive traits in this crop, and that organic fertilizers, in general, was not better than synthetic fertilizers in improving those traits. Interestingly, the response of the squash and indirectly on their legitimate floral visitors to the increase of temperature and the input of fertilizers vary widely among seasons, suggesting great temporal variation in plant-pollinator responses to temperature and nutrient availability, which makes food security more unpredictable.

## 1 Introduction

Climate change causes greater climate variability, affecting crop production and, thus food provision worldwide (Tubiello et al., 2007; Lobell et al., 2011; Ochieng et al., 2016). Particularly, the global temperature increase expected in the short term (i.e., 1.2−1.8°C from 2021 to 2040; IPCC, 2022) is expected to affect the physiology, phenology, and performance of various plants (Peñuelas and Filella, 2001; Hedhly et al., 2009; Schweiger et al., 2010; Prasad et al., 2008). For instance, it has been found that the increase in temperature rises the abortion of flowers and modifies the size of their reproductive organs (Saavedra et al., 2003; Sato et al., 2006; Scaven and Rafferty, 2013; Gérard et al., 2020; Akter and Klečka, 2022), decreases nectar quantity and quality (Petanidou and Smets, 1996; Keasar et al., 2008; Mu et al., 2015; Takkis et al., 2015; Descamps et al., 2018; Akter and Klečka, 2022), and reduces pollen viability (Fahad et al., 2015; Descamps et al., 2018). This in turn, may decrease the number of visits and richness of floral pollinators, resulting in lower fruit and seed production (Schweiger et al., 2010; Hoover et al., 2012; Scaven and Rafferty, 2013; Kerr et al., 2015).

On the other hand, different studies have shown that the input of nutrients into the soil increase the production and size of flowers, lengthen the flowering period, and improve the quality and quantity of nectar (Lau and Stephenson, 1993; Gardener and Gillman, 2001; Muñoz et al., 2005; Burkle and Irwin, 2010; Hoover et al., 2012; Gao et al., 2021). Given that pollinators show a greater preference for plants that have a greater floral display (Mitchell et al., 2004; Akter and Klečka, 2022), larger flowers (Conner and Rush, 1996) or flowers with better quality in rewards (Cnaani et al., 2006); the addition of soil fertilizers should benefit the interaction of plants with their pollinators, and thus, their reproductive success (Campbell and Halama, 1993; Hoover et al., 2012; Fernandez et al., 2019). For example, plants of *Ipomopsis aggregata* that received fertilizer produced larger flowers and more nectar, had longer flowering periods and received more visits than plants that received no supplementary fertilizer (Burkle and Irwin, 2010), while plants of *Chuquiraga oppositifolia* exposed to nitrogen enrichment produced more flowers than control plants, which benefited the frequency of pollinator visits (Muñoz et al., 2005).

Despite these benefits, adding nutrients to the soil through synthetic fertilizers, caused major environmental problems such as water pollution and soil degradation (Armenta-Bojórquez et al., 2010; Lu and Tian, 2017). Alternatively, organic fertilizers besides providing nutrients, also improves the physical characteristics of the soil (Moreno Reséndez et al., 2008), stimulates the presence of beneficial microorganisms (Ferreras et al., 2015), and increases the resistance to temperature stress (Fahad et al., 2015; Carreto-Morales et al., 2021) without causing adverse effects on the environment. Specifically, the vermicompost is an organic fertilizer obtained from the transformation of different organic wastes by the activity of earthworms and microorganisms (Moreno Reséndez et al., 2008; Domínguez, 2018), that provides several nutrients to the soil, improves porosity, allows greater moisture retention, promotes the presence of beneficial microorganisms (Moreno Reséndez et al., 2005; Bachman and Metzger, 2008; Ramos Oseguera et al., 2019), and improves some aspects of plant development (Hashemimajd et al., 2004; Ramos Oseguera et al., 2019), including traits associated with pollinator visitation (Cardoza et al., 2012; Jusselme et al., 2019). For instance, cucumber plants grew in soils enriched with vermicompost showed heavier flowers than control plants (Cardoza et al., 2012), while *Hylotelephium maximum* produces greater volume of nectar with higher concentration and receives a greater number of visits from pollinators in substrates enriched with vermicompost than in control plants (Jusselme et al., 2019).

Even though organic fertilizers can improve plant growth and fitness, its effects on floral biology, pollinator visits, and reproductive success in the face of increased temperatures in cultivated plants are still unknown, including the squash, *Cucurbita pepo*, which is a species of great economic importance cultivated throughout the world (Hoover et al., 2012). In this study, we experimentally compared the effect of organic and synthetic fertilizers on plant height, leaf area, number of leaves, days to flowering, number of flowers, the proportion of pistillate flowers, flower size, volume and concentration of nectar, visitation rate of legitimate floral visitors, visit duration, fruit set and number of seeds per fruit under a scenario of increased temperature in the squash. This species being completely dependent on pollinators to set fruit (Shuler et al., 2005), is expected to be particularly susceptible to climate change, as seen in several pollinator-dependent species (e.g., Kevan and Viana, 2003; Kjøhl et al., 2011).

Specifically, we asked the following questions: (1) Does the increase in ambient temperature affect the vegetative and floral traits, pollinator attraction, and reproductive success of squash? If so, (2) can adding nutrients mitigate the adverse effects of increased temperature on these traits? And, (3) are there any differences in these effects between plants that received organic and synthetic fertilizers? We predict that an increase in ambient temperature will negatively affect the vegetative and floral traits of squash, causing a decrease in the attraction of pollinators and, thus, a reduction in the reproductive success of the crop. Furthermore, we expect that adding nutrients will lessen these adverse effects caused by the increase in temperature and that plants with organic fertilizers will respond better than plants with synthetic fertilizers in reducing these effects.

## 2 Materials and methods

### 2.1 Experimental site

An agricultural plot in Tecpan de Galeana, Guerrero, Mexico (17°13’01.7’’ N, 100°38’38.1’’ W) was used as an experimental site. The area has a warm subhumid climate (Aw), an average annual temperature of 26.6°C, with a maximum of 32°C in April-May and a minimum of 18°C in December-January, an average annual precipitation of 1100 mm with a rainy period from June to November (precipitation ≈ 950 mm), and a dry period from December to May (precipitation < 70 mm) and an altitude of 50 masl (INEGI, 2009).

### 2.2 Study species

*Cucurbita pepo* L. is an herbaceous monoecious annual plant, with large flowers (Cerón-González et al., 2010) that open from the morning until noon (Campbell et al., 2013; Devi et al., 2015). Male flowers appear first and depend on insects to transport their pollen to female flowers (Shuler et al., 2005; Cerón-González et al., 2010). The temperature for optimum development oscillates between 18 to 28°C and shows a preference for hot humid places (Salehi et al., 2019). The addition of nutrients is an important factor in its growth (Castagnino et al., 2007), significantly affecting the yield and quality of the fruit (Rodas-Gaitán et al., 2012). In this study, commercial seeds of squash var. zuccini were used (Distribuidora Rancho Los Molinos S.A. DE C.V., Tepoztlan, Morelos, México).

### 2.3 Experimental design

To evaluate the combined effects of temperature and fertilizers on vegetative and floral traits, pollinator visitation, and reproductive success of *C. pepo*, a completely randomized experimental design was used in a 2x3 factorial arrangement. The factors considered were: (1) temperature (ambient and increased temperature) and (2) fertilizers(organic, synthetic, and without fertilizers). Considering the variation in the composition of pollinators between the dry season and the rainy season (Delgado-Carrillo et al., 2018), the experiment was carried out in both seasons of the year 2020 (dry season: April−June; rainy season: August−October). The six treatments were repeated 20 times (120 plants for each season).

**Figure 1:**
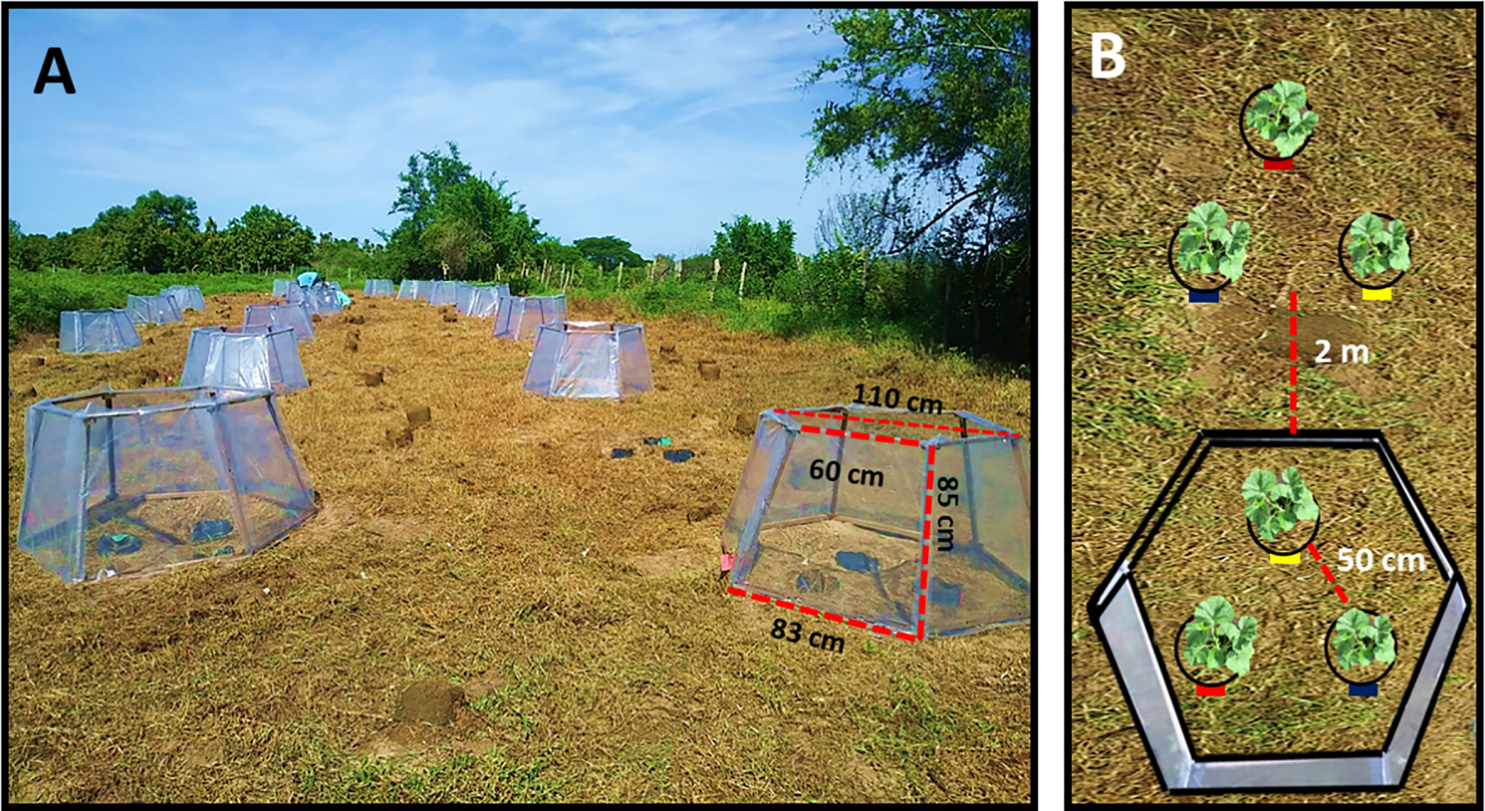
Field site showing **(A)** dimensions of OTCs where plants at increased temperature were placed and location of plants at ambient temperature (green oval), and **(B)** location of plants without fertilizer (yellow), with organic (red) and synthetic (blue) fertilizers inside and outside OTCs.

To increase the temperature of sown plants, twenty open top chambers (OTCs) with a wooden structure and transparent polyethylene (600 gauges, **Figure 1A**) were used. These OTCs have been widely used to study the response of plants to climate change in different ecosystems, increasing the ambient temperature between 0.7 and 4°C (Aerts et al., 2004; Klanderud, 2005; Ren et al., 2010). Throughout the experiment, the temperature was recorded every day at one-hour intervals with an automatic temperature and humidity recorder (HOBO MX2301, Onset Computer Corporation, MacArthur Blvd, Bourne, MA 02532, USA) placed at 10 cm above the ground inside and outside the OTCs. The distance between each arrangement (with OTC and without OTC) was two meters, each one with three plants (one from each fertilizer treatment, marked with different colored labels), with a distance between plants of 50 cm (**Figure 1B**). All plants received 1.5 L of water daily, except on rainy days.

Organic fertilizer consisted of 500 g of vermicompost (Fernatol^®^, Alchichica, Puebla, 73998, México) added per plant five days before sowing. In contrast, synthetic fertilizer was applied by using 30 g of a commercial product (Blaukorn Classic, Campo Expert, Zapopan, Jalisco, 45239, Mexico) per plant 15 days after sowing (DAS).

The seeds were sown on April 14 (dry season) and August 14 (rainy season) in a 35 x 35 cm black polyethylene bag. The bags were filled with 5 kg of agricultural soil from the area, homogeneously mixed, and randomly distributed in the bags, which were buried in the soil at a depth of 30 cm. Two squash seeds were placed in each bag and were randomly assigned to the fertilizer treatments. Seedling germination started at 5 DAS, and the true leaves appeared at 10 DAS. At that moment, the shortest plant was removed, leaving only one seedling per bag.

### 2.4 Vegetative and floral traits

The effect of increased temperature and fertilizer on vegetative traits was evaluated by measuring the following response variables: (1) height of the plant, (2) number of leaves per plant, and (3) leaf area of the largest leaf of each plant. Plant height was measured with a flexometer (Truper^®^, FA-5HD, precision: 0.1 cm) from the base of the stem to the highest leaf of each plant. The leaf area was estimated with ImageJ software (http://imagej.nih.gov/ij/) from a photography of the leaf taken with a 13-megapixel camera (LG, Q6 Plus), placed perpendicular to the leaf on a white background with a millimeter scale. All measurements were made at 30 DAS.

To evaluate the effect of the treatments on the floral biology of *C. pepo*, the following response variables were measured: (1) days to flowering − the number of days after germination until the first staminate and pistillate flower appeared, (2) number of flowers − staminate and pistillate flowers throughout the flowering stage, (3) proportion of pistillate flowers − number of pistillate flowers/total number of flowers, (4) corolla diameter, (5) floral tube diameter, (6) floral tube length, (7) petal width (of the largest petal), (8) length of stamens, (9) length of the style-stigma, (10) nectar volume, and (11) nectar concentration (**Supplementary Figure 1**). Sample sizes vary due to the availability of daily flowers (range: 1-3 flowers and 2-7 flowers per plant, respectively for pistillate and staminate flowers). A digital caliper (Mitutoyo Corp., CD-8” ASXL, Kanagawa, Japan, precision: 0.01 mm) was used for size measurements. Nectar volume and concentration were measured every hour from 6:30 to 10:30 h in flowers randomly selected and bagged prior to anthesis (i.e., 5:30 h) using respectively, graduated microcapillary tubes and a digital refractometer (Atago, Master-53α, Tokyo, Japan, precision: 0.5%).

### 2.5 Legitimate floral visitors

The effect of temperature and fertilizerson the attraction of legitimate floral visitors was studied by filming (Sony HDR-CX455) on sunny days, single staminate and pistillate flowers of *C. pepo*. The video recordings in the flowers were made from 8:00 to 09:00 and from 7:30 to 8:30 h in the dry and the rainy season, respectively, which corresponds to the peak foraging times of most floral visitors according to preliminary data (**Supplementary Figure 2**). The number of hours filmed varied among seasons due to flower availability – rainy season: 110 hours (6-14 hours per treatment), dry season: 74 hours (3-10 hours per treatment). For each floral visit three variables were recorded: (1) species of floral visitor (at the lowest possible taxonomic level), (2) visit duration (in seconds), and (3) contact with the reproductive organs of the flower. The relative importance of each species of floral visitor was estimated as the number of visits per their frequency of contact with stamens and stigma, standardized to percentages in order to compare across all legitimate species. Only visitors contacting the flower’s reproductive organs were considered for analysis. Three individuals of each floral visitor were collected for identification using the bee species guide and world checklist (Asher and Pickering, 2020). During filming, the OTCs were removed prior the aperture of the flower until the flowers began to wilt to avoid any possible bias in visitation rates among treatments, since OTCs could act as barriers to pollinator movements (i.e., Adamson and Iler, 2021) and flowers from plants with OTCs may have more floral rewards to visitors than plants at ambient temperature.

### 2.6 Reproductive success

The fruit set and number of seeds were estimated to test the effect of the increase of nutrients and temperature on the reproductive success of the plant. Fruit set was only estimated in the pistillate flowers of plants from which the OTC was removed from anthesis to flower wilting. Five days after flowering, all pistillate flowers were checked to test for fruit formation. Fruits produced were harvested ten days after floral opening, and the number of mature seeds was counted.

### 2.7 Statistical analysis

Considering that the floral traits (corolla diameter, floral tube diameter, floral tube length, petal width and length of stamens, or length of style-stigma) measured in pistillate and staminate flowers were significantly correlated in both seasons (r > 0.140, p < 0.05 in all comparisons; **Supplementary Figures 3–6**), only the diameter of the corolla was used in further analyses as an indicator of the size of the flower. Two-way analysis of variance (ANOVA) was performed to analyze the effects of temperature, fertilizers, and their interactions on plant height, nectar volume, and sugar concentration in the dry season and corolla diameter for both seasons since data show normal distribution and homoscedasticity. Generalized linear models (GLM) were performed with a Poisson error distribution and a logarithmic link function to compare the number of leaves, the number of days to flowering, the number of flowers, the number of visits of legitimate floral visitors, and the number of seeds per fruit among treatments. For the duration of the visits, a GLM was performed with a negative binomial distribution due to overdispersion. Since each flower received more than one visit and not all came from a different flower or plant, the data for the duration of the visits included pseudoreplications. For plant height, nectar volume, and sugar concentration in the rainy season and leaf area for both seasons, a GLM was performed with a gamma distribution and a logit link function. For the fruit set, a GLM was performed with a binomial distribution and a logit link function, whereas for the proportion of pistillate flowers, the data were transformed with the square root and then analyzed with GLM with a Gaussian distribution. When floral traits were measured in more than one flower per plant, these values were averaged to obtain a value per plant. All analyzes were performed with InfoStat software (InfoStat version 2020, Centro de Transferencia InfoStat).

## 3 Results

During the dry season, an average temperature of 28.08 and 29.64°C were recorded in the ambient and within the OTC, respectively, and a daily average increase of 1.56°C (**Supplementary Figure 7A**). In the rainy season, an average temperature of 28.51 and 30.02°C were recorded in the ambient and within the OTC, respectively, and a daily average increase of 1.51°C (**Supplementary Figure 7B**).

In general, our results indicate that the increase in temperature and fertilizers impact significantly several vegetative and floral traits, as well as their interaction with their legitimate floral visitors and the reproductive success of the squash at both seasons (**Supplementary Tables 1** and **2**). In the following paragraphs we explain specifically how these factors affect each variable.

### 3.1 Vegetative traits

**Figure 2:**
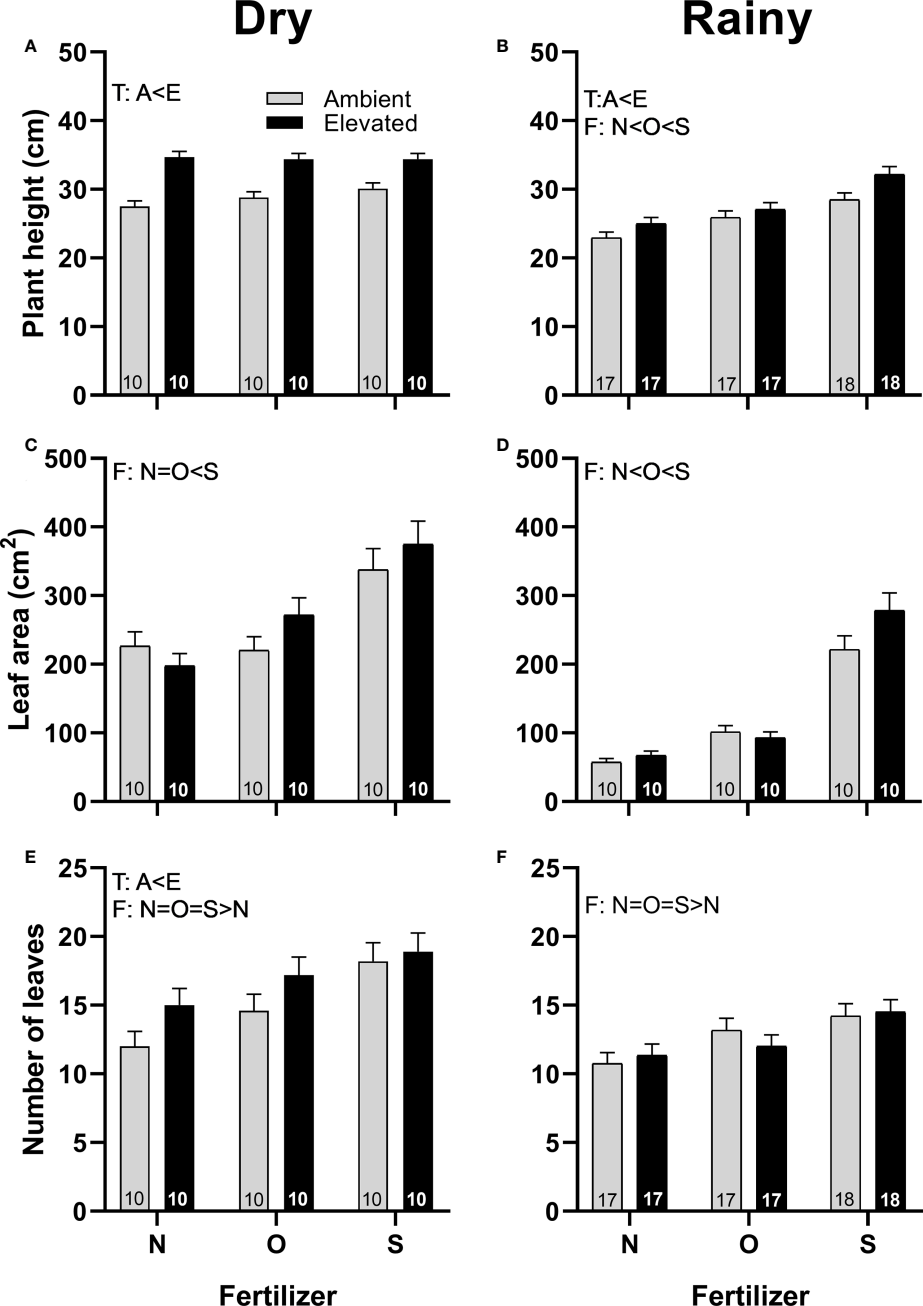
Effects of the increase in temperature (T) and addition of fertilizer (F) on plant height **(A, B)**, leaf area **(C, D)**, and the number of leaves produced per plant **(E, F)** of *C. pepo* during the dry (left) and the rainy (right) season (mean ± SE; n: number inside bar). Significant differences in the main factors (T or F) are represented with capital letters (A, ambient temperature; E, elevated temperature; N, no supplementary fertilizer; O, organic fertilizer; S, synthetic fertilizer). Mean and standard deviation values are in **Supplementary Tables 1**, **2** and the results of the statistical tests are summarized in **Supplementary Table 3**.

Squash plants under increased temperature grew taller in both seasons (**Figures 2A, B**) and produced more leaves in the dry season (**Figure 2E**) than plants at ambient temperature. Plants that received synthetic fertilizers grew taller in the rainy season (**Figure 2B**), and produced larger leaves in both seasons than plants grown with organic fertilizer or without fertilizer (**Figures 2C, D**), and produced more leaves than control plants in both seasons (**Figures 2E, F**). Plants grown with organic fertilizer produce taller plants (**Figure 2B**) and larger leaves in the rainy season (**Figure 2D**) than control plants. No interaction for both factors was detected (**Supplementary Table 3**).

### 3.2 Floral traits

#### 3.2.1 Pistillate flowers

**Figure 3:**
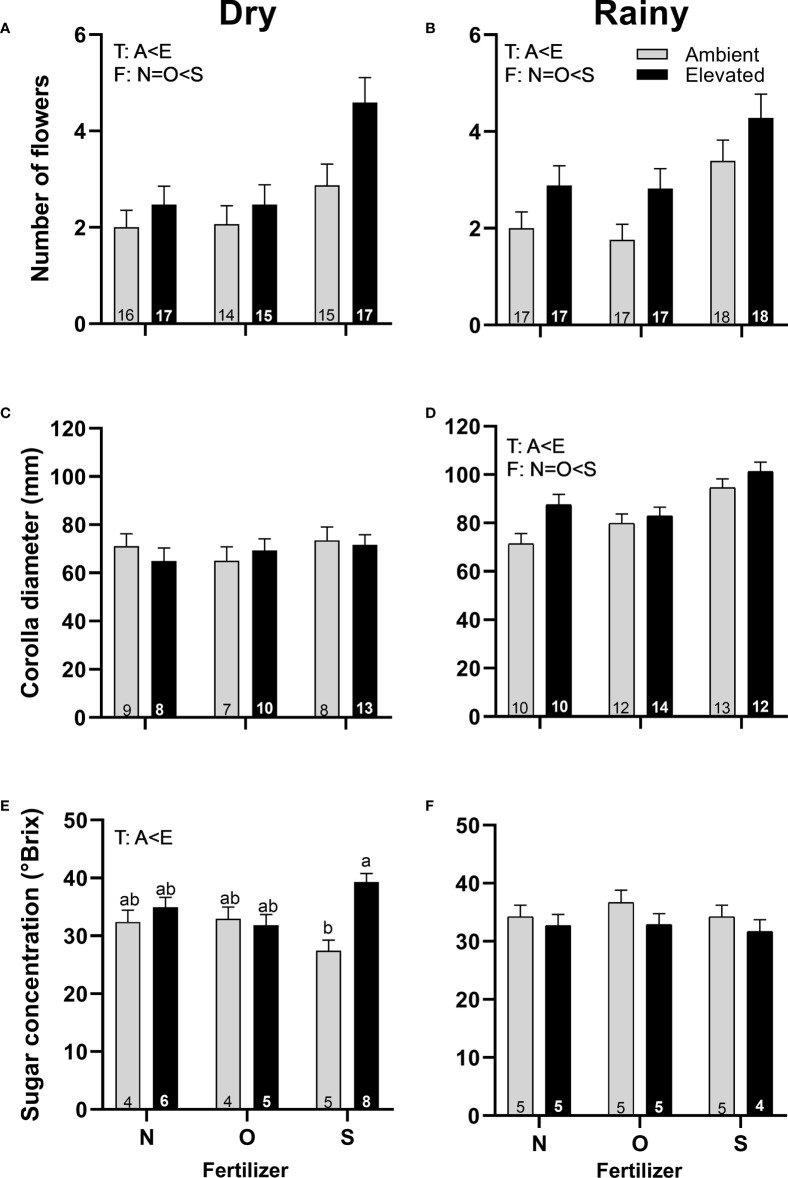
Effects of the increase in temperature (T) and addition of fertilizer (F) on the number of flowers **(A, B)**, corolla diameter **(C, D)** and sugar concentration **(E, F)** of pistillate flowers of *C. pepo* during the dry (left) and the rainy (right) season (mean ± SE; n: number inside bar). Significant differences in the main factors (T or F) are represented with capital letters (A, ambient temperature; E, elevated temperature; N, no supplementary fertilizer; O, organic fertilizer; S, synthetic fertilizer). Different lower-case letters above bars indicate statistically significant T x F interactions. Mean and standard deviation values are in **Supplementary Tables 1**, **2** and the results of the statistical tests are summarized in **Supplementary Table 3**.

Plants in increased temperature produced more pistillate flowers in both seasons (**Figures 3A, B**), were greater in size in the rainy season (**Figure 3D**), and produced nectar more concentrated in the dry season (**Figure 3E**) than plants at ambient temperature. Plants with synthetic fertilizers produce more pistillate flowers in both seasons (**Figures 3A, B**) and bigger flowers in the rainy season (**Figure 3D**) than plants with organic fertilizer or without fertilizer. A significant interaction between temperature and fertilizer was found in the sugar concentration of nectar of pistillate flowers in the dry season (**Figure 3E**; **Supplementary Table 3**). No differences were found for pistillate flowers in corolla diameter (**Figure 3C**) or sugar concentration (**Figure 3F**) in the rainy season, in the timing of flowering or nectar volume among treatments in any season.

#### 3.2.2 Staminate flowers

**Figure 4:**
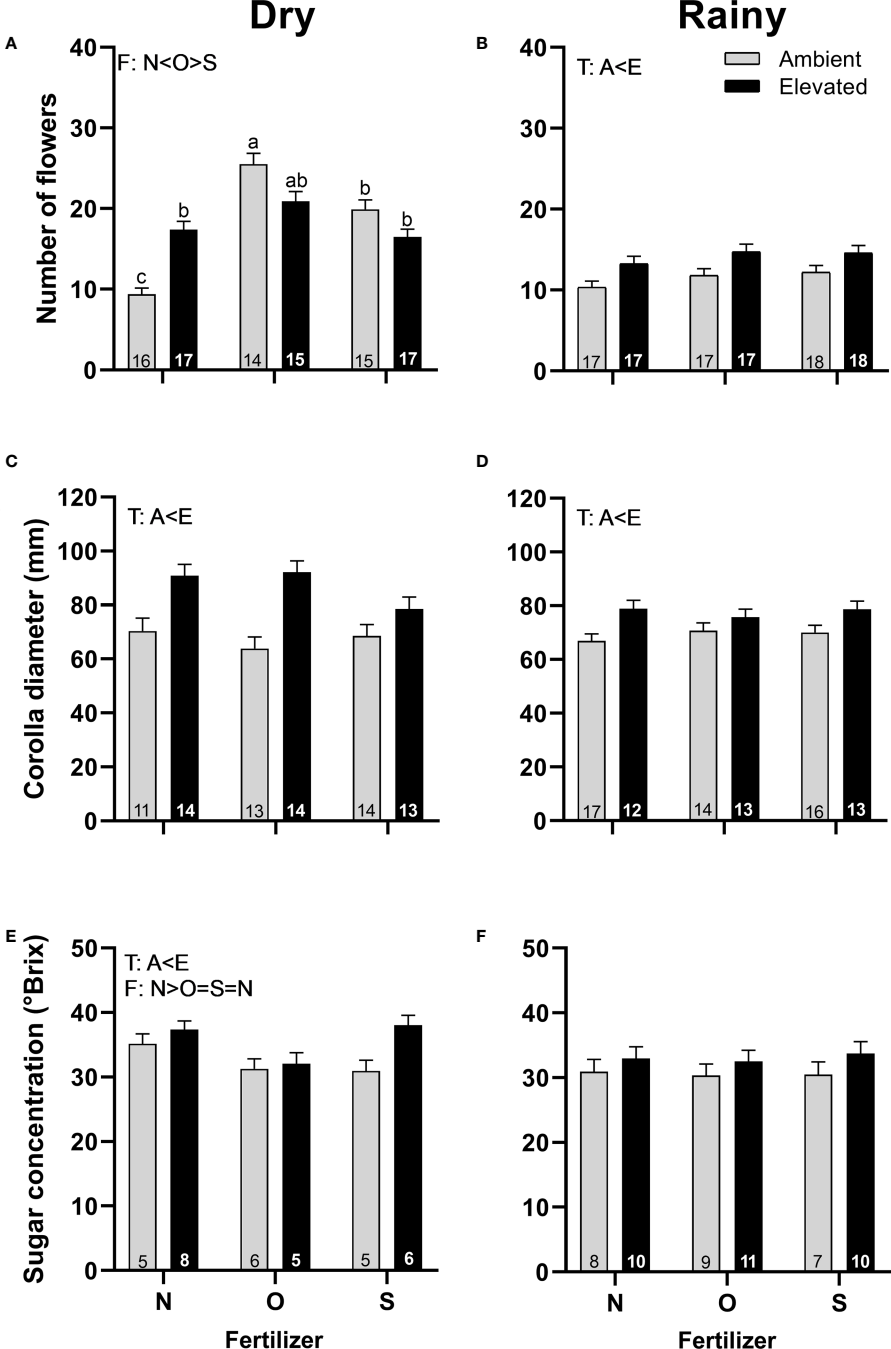
Effects of the increase in temperature (T) and addition of fertilizer (F) on the number of flowers **(A, B)**, corolla diameter **(C, D)**, and sugar concentration **(E, F)** of staminate flowers of *C. pepo* during the dry (left) and the rainy (right) season (mean ± SE; n: number inside bar). Significant differences in the main factors (T or F) are represented with capital letters (A, ambient temperature; E, elevated temperature; N, no supplementary fertilizer; O, organic fertilizer; S, synthetic fertilizer). Different lower-case letters above bars indicate statistically significant T x F interactions. Mean and standard deviation values are in **Supplementary Tables 1**, **2** and the results of the statistical tests are summarized in **Supplementary Table 3**.

Plants in OTC produced more staminate flowers in the rainy season (**Figure 4B**), flowered earlier in the dry season (**Supplementary Tables 1**, **3**), were greater in size in both seasons (**Figures 4C, D**), and produced nectar with higher concentration in the dry season (**Figure 4E**) than plants at ambient temperature. In the dry season, plants with organic fertilizer produced more male flowers than plants with synthetic fertilizers or without fertilizers (**Figure 4A**) but produced nectar with a lower concentration than flowers without fertilizers (**Figure 4E**). A significant interaction between temperature and fertilizer occurred in the dry season in the number of flowers produced per plant (**Figure 4A**; **Supplementary Table 3**). On the other hand, no differences were found in the concentration of sugar in nectar in the rainy season (**Figure 4F**), the timing of flowering, nectar volume, or nectar concentration among treatments in the rainy season.

#### 3.2.3 Flower sex ratio

**Figure 5:**
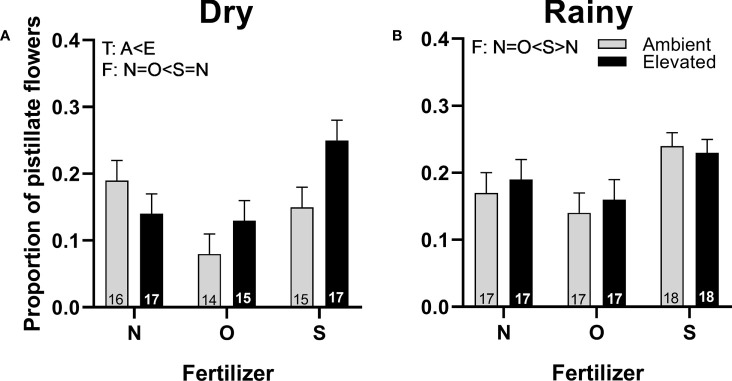
Effects of the increase in temperature (T) and addition of fertilizer (F) on the proportion of pistillate flowers of (*C*) *pepo* during the dry **(A)** and the rainy season **(B)** (mean ± SE; n: number inside bar). Significant differences in the main factors (T or F) are represented with capital letters (A, ambient temperature; E, elevated temperature; N, no supplementary fertilizer; O, organic fertilizer; S, synthetic fertilizer). Mean and standard deviation values are in **Supplementary Tables 1**, **2** and the results of the statistical tests are summarized in **Supplementary Table 3**.

The proportion of pistillate flowers was higher in plants with increased temperature in the dry season (**Figure 5A**) than in plants at ambient temperature. Plants with synthetic fertilizer showed an increase in the proportion of pistillate flowers compared to plants with organic fertilizer in both seasons (**Figures 5A, B**) and with plants that receive no supplementary fertilizer in the rainy season (**Figure 5B**). No interaction for both factors was detected (**Supplementary Table 3**).

### 3.3 Legitimate floral visitors

During 184 hours of filming, we observed a total of 1473 visits to the flowers of *C. pepo*, corresponding to eight species or morphospecies of insects (**Supplementary Tables 4**, **5**). Of these, 1029 legitimate visits (i.e., those that contact anthers and stigma) were recorded; 422 of them were observed in the dry season, corresponding mainly to three taxa: honeybee (*Apis mellifera*, 179 visits: ~4 visits/flower/h), sweat bees (Halictid bees, 137 visits: ~2 visits/flower/h), and carpenter bees (*Xylocopa*, 87 visits: ~1 visit/flower/h); whereas in the rainy season, 607 legitimate visits were recorded, being the squash bees, *Peponapis*, the most dominant visitor (429 visits: ~4.5 visits/flower/h), followed by sweat bees (134 visits: ~1.5 visits/flower/h), and *A. mellifera* (44 visits: ~0.5 visits/flower/h). The most important legitimate visitors in the dry season were the sweat bees (74%), followed by the carpenter bees (23%) and honeybees (2%), whereas in the rainy season were the squash bees (76%), followed by the sweat bees (12%) and honeybees (6%; **Supplementary Tables 4**, **5**).

**Figure 6:**
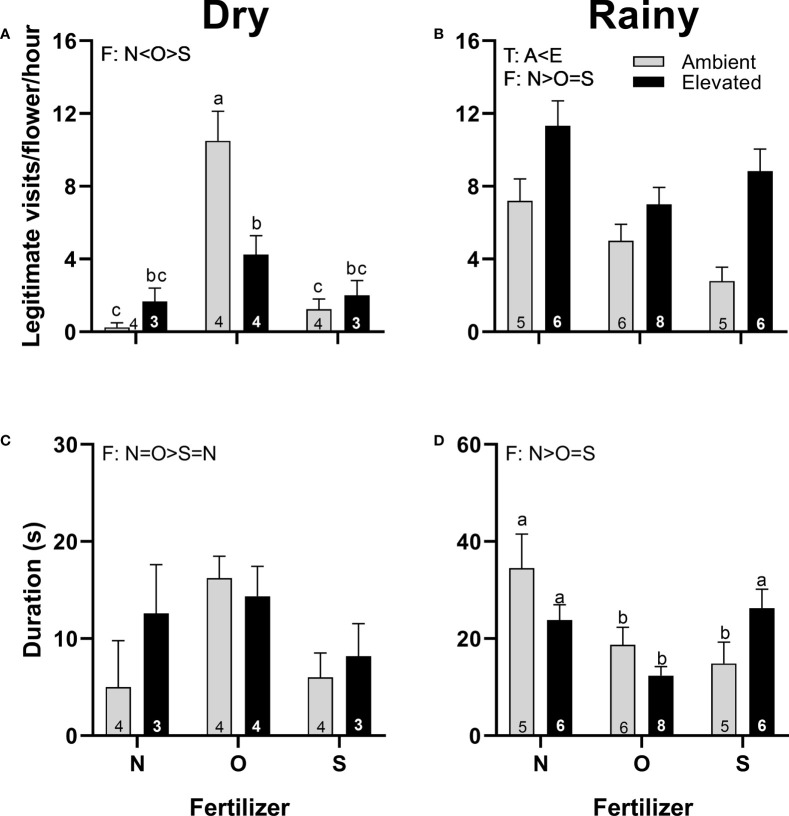
Effects of the increase in temperature (T) and addition of fertilizer (F) on legitimate floral visits per flower per hour **(A, B)** and visit duration **(C, D)** in pistillate flowers of (*C*) *pepo* during the dry (left) and the rainy (right) season (mean ± SE; n: number inside bar). Significant differences in the main factors (T or F) are represented with capital letters (A, ambient temperature; E, elevated temperature; N, no supplementary fertilizer; O, organic fertilizer; S, synthetic fertilizer). Different lower-case letters above bars indicate statistically significant T x F interactions. Mean and standard deviation values are in **Supplementary Tables 1**, **2** and the results of the statistical tests are summarized in **Supplementary Table 3**.

Pistillate flowers of plants at increased temperature received more visits than the flowers of the plants at ambient temperature in the rainy season (**Figure 6B**). However, the duration of visits was not affected by the increase in temperature in both seasons (**Figures 6C, D**). In the dry season, female flowers from plants that received organic fertilizer received more visits (**Figure 6A**), and visitors lasted longer than flowers from plants that received synthetic fertilizer (**Figure 6C**). Contrarily, in the rainy season, pistillate flowers from plants that receive no supplementary fertilizer received more visits (**Figure 6B**), and visitors lasted longer (**Figure 6D**) than flowers from plants that received fertilizer. A significant interaction between temperature and fertilizer occurred in the number of legitimate visits in the dry season (**Figure 6A**) and in the duration of visits in the rainy season (**Figure 6D**).

**Figure 7:**
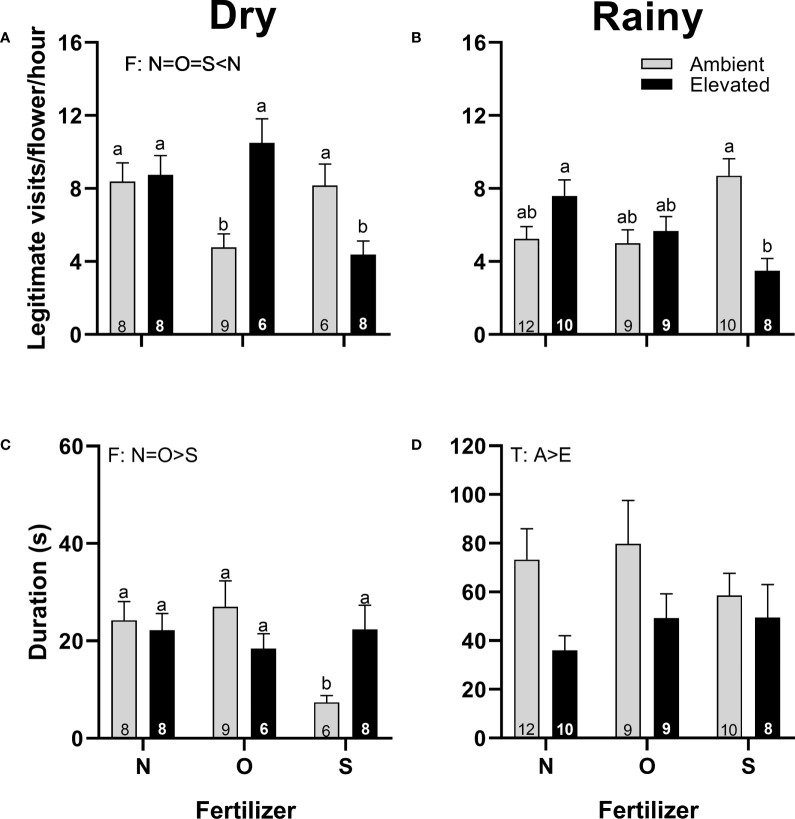
Effects of the increase in temperature (T) and addition of fertilizer (F) on legitimate floral visits per flower per hour **(A, B)** and visit duration **(C, D)** in staminate flowers of (*C*) *pepo* during the dry (left) and the rainy (right) season (mean ± SE; n: number inside bar). Significant differences in the main factors (T or F) are represented with capital letters (A, ambient temperature; E, elevated temperature; N, no supplementary fertilizer; O, organic fertilizer; S, synthetic fertilizer). Different lower-case letters above bars indicate statistically significant T x F interactions. Mean and standard deviation values are in **Supplementary Tables 1**, **2** and the results of the statistical tests are summarized in **Supplementary Table 3**.

In both seasons, staminate flowers of plants at increased temperature received a similar number of visits (**Figures 7A, B**), but were shorter in duration than flowers from plants at ambient temperature in the rainy season (**Figure 7D**). In the dry season, staminate flowers from plants with synthetic fertilizer received fewer visits (**Figure 7A**), and visitors lasted less (**Figure 7C**) than staminate flowers from plants that receive no supplementary fertilizer; while in the rainy season, fertilizer did not affect the number of visits or duration (**Figures 7B, D**). There was an interaction (T x F) in the number of legitimate visits in both seasons and in the duration of visits in the dry season (**Figures 7A−C**; **Supplementary Table 3**).

### 3.4 Reproductive success

**Figure 8:**
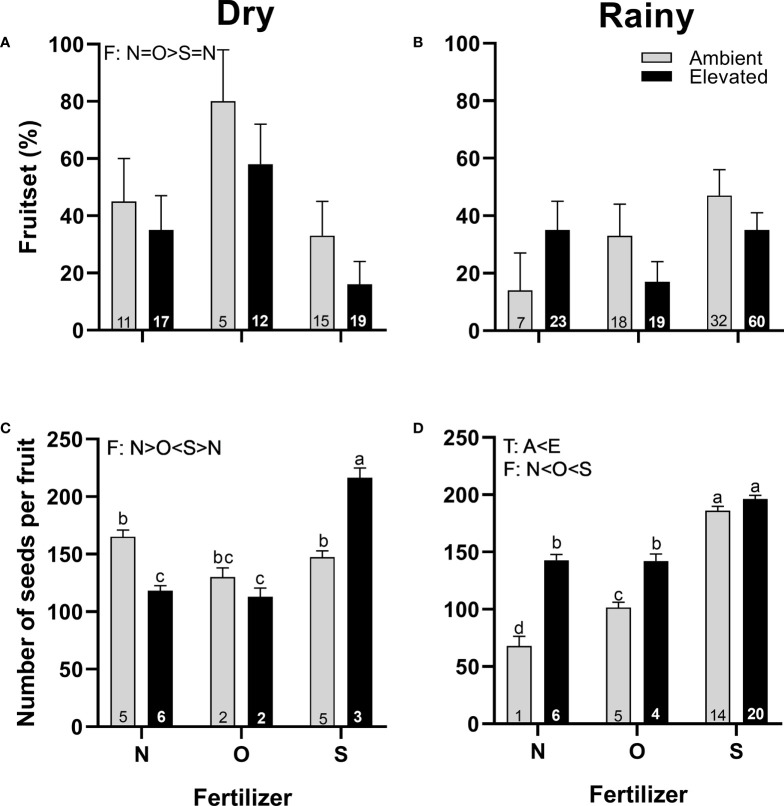
Effects of the increase in temperature (T) and addition of fertilizer (F) on fruit set **(A, C)** and the number of seeds per fruit **(B, D)** of *C. pepo* during the dry (left) and the rainy (right) season (mean ± SE; n: number inside bar). Significant differences in the main factors (T or F) are represented with capital letters (A, ambient temperature; E, elevated temperature; N, no supplementary fertilizer; O, organic fertilizer; S, synthetic fertilizer). Different lower-case letters above bars indicate statistically significant T x F interactions. Mean and standard deviation values are in **Supplementary Tables 1**, **2** and the results of the statistical tests are summarized in **Supplementary Table 3**.

Even when the increase in temperature does not alter the fruit set in any season (**Figures 8A, B**), it did increase the number of seeds produced per fruit in the rainy season compared to ambient temperature (**Figure 8D**). On the other hand, adding organic fertilizer during the dry season increased the fruit set compared to synthetic fertilizer (**Figure 8A**), but the number of seeds per fruit was the lowest compared to the other treatments (**Figure 8C**). The number of seeds per fruit was lowest in plants that receive no supplementary fertilizer in the rainy season, while plants with synthetic fertilizer produced more seeds than plants with organic fertilizer (**Figure 8D**). A significant interaction between temperature and fertilizer was detected in the production of seeds in both seasons (**Figures 8C, D**; **Supplementary Table 3**).

## 4 Discussion

**Figure 9:**
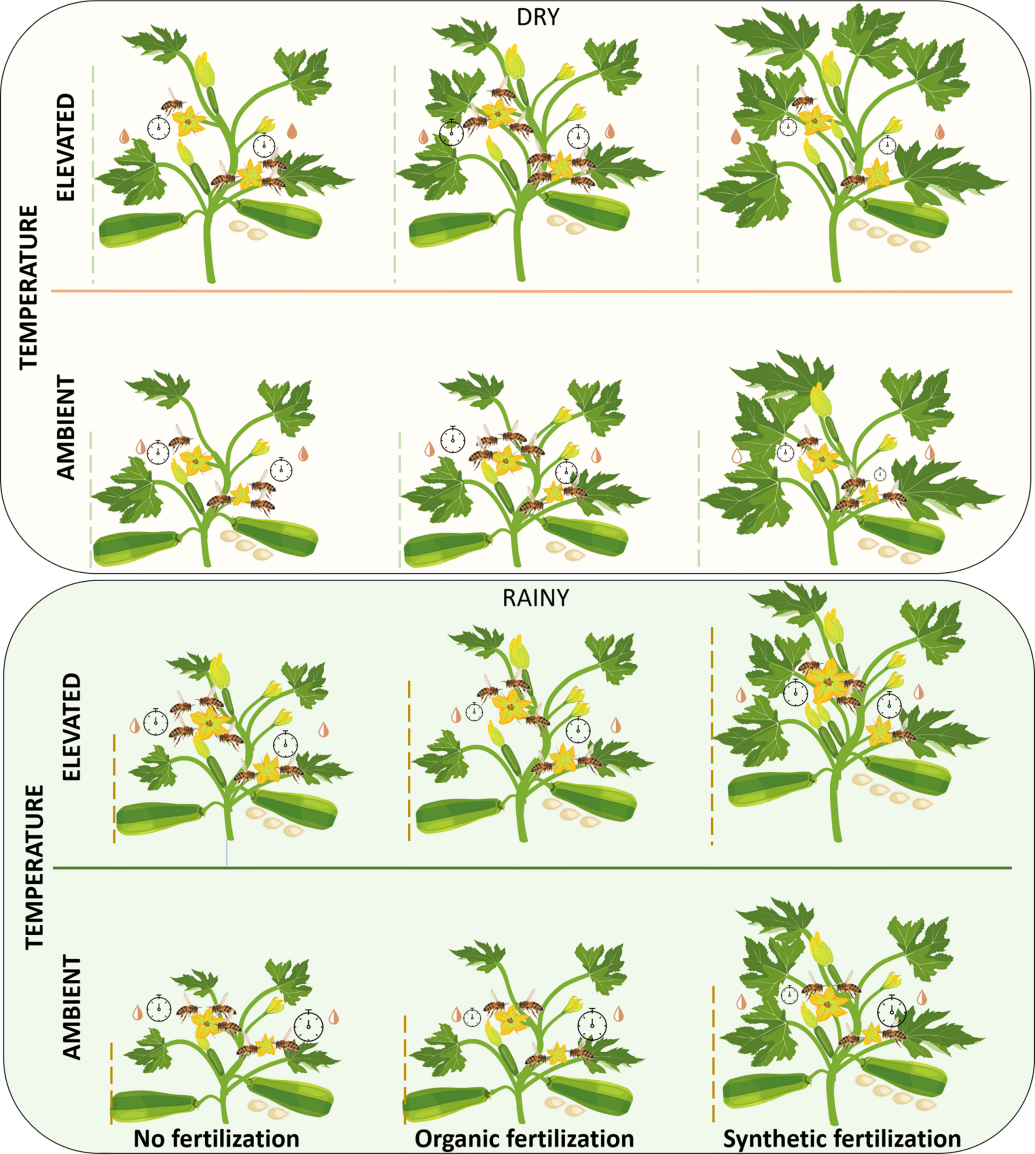
Schematic representation of increased temperature and fertilizer impacts on vegetative and floral traits, legitimate floral visitors, and reproductive success of *C. pepo* plants grown in the dry (top) and rainy (bottom) seasons. An increase in the size or number of parts indicates a significant increase in that variable. The dotted lines represent the height of the plants. The number and size of pistillate flowers are represented by 
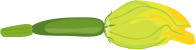
 and 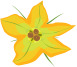, respectively. Number and size of staminate flowers are represented by 
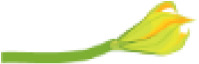
 and 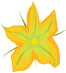, respectively. The volume and concentration of the nectar is represented by the symbol 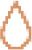, fuller symbols indicate greater concentration. The number of bees in each flower represents the visitation rate of legitimate floral visitors. The duration of the visits is represented by the size of the 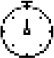. Fruit set is represented by the number of fruits on the plants and the number of seeds is represented by the number of seeds under the fruits. Nectar volume and concentration, visitation rate, and visitation duration are shown, respectively on the left and right side of each plant for pistillate and staminate flowers. No statistical comparisons were performed between pistillate and staminate flowers, nor between dry and rainy seasons. For specific analyses, data and statistical values see **Supplementary Tables 1**–**3** and **Figures 2**–**8**.

Using a squash model system during two seasons, we have demonstrated that climatic warming and fertilizers had significant impacts on vegetative and floral traits, as well as on their interaction with their pollinators, and consequently, on the reproductive success of this pollinator-dependent crop plant. Contrary to our predictions, we found that the increase in ambient temperature positively influenced several vegetative, floral, and reproductive traits of this vegetable, and that organic fertilizer, in general, was not better than synthetic fertilizer in improving those traits. Interestingly, we found great variation among seasons in the response of squash to increased temperature and the addition of fertilizers on several traits mainly on the attraction of legitimate floral visitors and the plant’s reproductive success (**Figure 9**).

In contrast to most studies (e.g., Saavedra et al., 2003; Aerts et al., 2004; Klanderud, 2005; Ren et al., 2010; Liu et al., 2012; Mu et al., 2015; Feeley et al., 2017; Adamson and Iler, 2021), our results indicate that a moderate rise in ambient temperature (~1.5°C) has positive effects on squash, including the height of the plant, number of leaves, number of pistillate and staminate flowers, flower size, nectar concentration, number of legitimate visits to pistillate flowers, number of seeds per fruit, and a reduction in the duration of visits to staminate flowers. Several studies have found that an increase in temperature (0.7 to 4°C), negatively affects the attractiveness of plants to their pollinators due to a decrease in vegetative growth, production and size of flowers, and the production and quality of nectar (Petanidou and Smets, 1996; Saavedra et al., 2003; Sato et al., 2006; Hoover et al., 2012; Liu et al., 2012; Scaven and Rafferty, 2013; Takkis et al., 2015; Mu et al., 2015; Descamps et al., 2018; Descamps et al., 2020; Akter and Klečka, 2022). Discrepancies with these studies may be because these have focused on plants from temperate regions (i.e., latitudes greater than 32°), where more drastic temperature changes are expected (Saavedra et al., 2003; Feeley et al., 2017). The response of temperate plants is different from tropical plants (Garruña-Hernández et al., 2014; Feeley et al., 2017) since the latter have a higher threshold temperature value than the former (Wahid et al., 2007; Driedonks et al., 2016). Heat sensitivity usually differs between crop types, being more tolerant those tropical cultivars adapted to higher temperatures than their temperate counterparts (Driedonks et al., 2016).

The increase in the number of leaves, leaf area and/or the size of plants exposed to higher temperatures or input of fertilizers may cause greater photosynthetic activity and an increase of photoassimilates that could explain the increase in the number and the size of flowers (Queiroga et al., 2007; Weiner et al., 2009; Han et al., 2011; Pôrto et al., 2012; Li et al., 2020). Nevertheless, an increase in the number of pistillate flowers due to an increase in temperature does not necessarily relate to higher fruit set. This may be because fruit set and seed production are also influenced by viability, availability, and compatibility of pollen deposited on stigmas (Sato et al., 2006; Borbolla-Perez et al., 2016). For example, when tomato was grown at moderately elevated temperatures, fruit set and pollen viability were reduced (Sato et al., 2006). Further studies are needed to test whether higher temperatures affect the pollen viability and deposition in the squash.

The increase of photoassimilates could also explain the higher concentration of sugar in nectar found in pistillate and staminate flowers at elevated temperatures. However, this only occurs during the dry season, probably due to the lesser amount of water from rain and the lower air humidity found in this season (dry season - 63.10% outside and 60.93% inside the OTC; rainy season - 82.22% outside and 78.30% inside the OTC).

On the other hand, as expected, the addition of fertilizers improves some vegetative (i.e., plant height, number of leaves, leaf area), floral (i.e., number of flowers, flower size), and reproductive traits (i.e., fruit set, number of seeds). Given that monoecious plants, such as squash, possess unisexual flowers, the effects of the addition of fertilizer may be different for the male and female function (Goldman and Willson, 1986; Dorken and Barrett, 2004; Hoover et al., 2012; Campbell et al., 2013; Li et al., 2020; Gao et al., 2021). For instance, the increase in the number of seeds may result from an increase in allocating resources from fertilizers to female function (Campbell and Halama, 1993). Given that the development of fruits and seeds requires a larger amount of resources compared to the male function (Goldman and Willson, 1986; Campbell et al., 2013), plants grew at elevated temperature and with fertilizer seems to invest more resources to female function (mainly number of seeds) than control plants (see Han et al., 2011; Li et al., 2020).

Similarly, plants that received fertilizer are expected to invest more resources in producing floral rewards such as pollen and nectar, especially those that depend on animals to set fruit. However, contrary to our expectations, we found that nectar volume did not vary with increasing temperature or fertilizer. Although nectar production often represents a considerable investment for plants (Southwick, 1984; Ashman and Schoen, 1997), several studies have shown that changes in nectar production are not always influenced by changes in resource availability (David et al., 2019). Instead, the age and sex of the flower, flowering period, and the position of the flower on the plant are some factors that can influence nectar production (Lu et al., 2015). Furthermore, given that plants with variable nectar production would be less preferred by pollinators (Biernaskie et al., 2002), it is likely that a constant production of nectar independently of nutrient conditions could be a common strategy to maintain constant visits by pollinators, even at the expense of lower production of other floral traits, such as the number or size of the flowers (Flacher et al., 2020). This could explain why plants that received fertilizer display more and bigger flowers but with similar nectar volume than plants that received no supplementary fertilizer.

The increase in the number and size of pistillate flowers caused by an increase in the ambient temperature in the rainy season seems to explain the increase in the number of legitimate visits. This could also explain the longer duration of visits in the rainy season to the fewer pistillate flowers from plants without supplementary fertilizer, since pollinators usually stay longer in the flowers in order to obtain as many nutritional resources as possible when the abundance of flowers is low (Ortiz-sánchez and Tinaut, 1994; Aguado et al., 2019). Similar to Chen et al. (2021), we found that organic fertilizer increased the number of visits (only in the dry season), probably because the organic matter of this manure contains a more diverse mix of micro and macronutrients compared to synthetic fertilizers; providing a greater amount of nutrients that could be assigned to improve floral attractiveness, favouring the visit of pollinators (Fernandez et al., 2019). Similarly, Cardoza et al. (2012) found that cucumber flowers treated with vermicompost received more visits from pollinators, probably because this fertilizer promotes volatile signals in flowers. In contrast, in the rainy season, pistillate flowers from plants with fertilizer received fewer visits by legitimate floral visitors but produced significantly more seeds than plants with no supplementary fertilizer. Although there is no doubt that the greater content of nutrients was necessary for appropriate seed development, it is unclear why flowers from plants with no supplementary fertilizer attracts more legitimate floral visitors than plants with fertilizer in the rainy season, despite their small number and size. Probably the more and bigger pistillate flowers from plants with synthetic fertilizer attracts many illegitimate floral visitors which could decrease the amount of rewards to legitimate visitors.

It is interesting to note that bees of the genus *Peponapis*, considered specialized pollinators of pumpkins (López-Uribe et al., 2016), were absent in the dry season when *A. mellifera* was the most common legitimate visitor and the sweat bees were the most important legitimate visitor, but was the most frequent and important legitimate visitor in the rainy season. Even when it has been reported that fruit set for pumpkin flowers visited two or fewer times by *Peponapis pruinosa* and *A. mellifera* were similar (Artz and Nault, 2011), we found that the exotic *A. mellifera*, despite being a common visitor, is not as important legitimate visitor as the sweat bees (in both seasons), the carpenter bees (in the dry season) or the squash bees (in the rainy season). Furthermore, this exotic honeybee could negatively influence the abundance and behaviour of native pollinators, as reported by several studies (Badano and Vergara, 2011; Layek et al., 2021). These results highlight the importance of species richness of native species rather than the abundance of common species in the provision of ecosystem services (but see Winfree et al., 2015) and that serves as an insurance to fruit production of this and several bee-dependent crops. Indeed, it is expected that crops pollinated by several species may be buffered against the effect of climate change (Rader et al., 2013). Thus, it is crucial to preserve the habitats of all pollinators to confer greater resilience to agroecosystems (Dainese et al., 2019), and to maintain food provision globally.

Interactions between temperature and fertilizer were rarely observed in both seasons, except in the attraction of pollinators (i.e., number and duration of visits) and the number of seeds per fruit. For instance, we found that the number of visits to pistillate flowers in the dry season was increased at ambient temperature and with the addition of organic fertilizers, whereas in the staminate flowers occur the opposite. We also found a decrease in the duration of visits at ambient temperature with synthetic fertilizer on pistillate and staminate flowers during the rainy and the dry season, respectively. Finally, the number of seeds produced per fruit was higher in plants that received synthetic fertilizer exposed to higher temperatures in both seasons, while the number of seeds per fruit was lower in plants with organic fertilizer and without supplementary fertilizer at ambient temperature in the rainy season and at elevated temperature in the dry season. These results indicate that the temperature and fertilizer will have complex and multiple effects on pollinators that we are just glimpsing and understanding.

It is outstanding that a small increase of 1.5°C in ambient temperature will significantly impact vegetative and floral traits and will have direct implications on reproductive success through the increase in seed production due to an increase in the visit of pollinators. However, it is important to note that these effects vary among seasons, suggesting high temporal variation in plant responses to temperature and nutrient availability. Even when tropical plants may be less vulnerable to higher temperatures than temperate plants, studies about the response of their interactors (mutualists and antagonists) to these changes are needed to understand whether plant species are prone to persist under climate change scenarios. Moreover, further studies should examine the effects of other main drivers of global change (i.e., nitrogen deposition, increasing atmospheric carbon dioxide, water availability; see Hoover et al., 2012; Akter and Klečka, 2022) on main crops to have a better vision of what the future holds in terms of food security.

## Data availability statement

The datasets presented in this study can be found in online repositories. The names of the repository/repositories and accession number(s) can be found below: https://doi.org/10.6084/m9.figshare.20224401.v4 Figshare.

## Author contributions

ML-A, VR-G, and LA-F conceived the ideas. ML-A, RL-G, VR-G, and LA-F contributed to the design of the methodology. ML-A and RL-G collected the data. ML-A and RL-G processed and analyzed the data. ML-A wrote the first draft of the manuscript with inputs from RL-G and VR-G. VR-G, LA-F, JK-D, and GH-F supervised the investigation. All authors contributed critically to the drafts and gave final approval for publication.

## Acknowledgments

The authors would like to thank four reviewers for insightful comments that significantly improved this manuscript. We thank J. A. Gama-Salgado, O. López-Salas, F. Severiano-Galeana, and C. de la O, who helped build the OTCs and set up the experiment. The first author thanks the National Council of Science and Technology (CONACYT) for the scholarship granted for her master’s studies (CVU: 1005173).

## Conflict of interest

The authors declare that the research was conducted in the absence of any commercial or financial relationships that could be construed as a potential conflict of interest.

## Publisher’s note

All claims expressed in this article are solely those of the authors and do not necessarily represent those of their affiliated organizations, or those of the publisher, the editors and the reviewers. Any product that may be evaluated in this article, or claim that may be made by its manufacturer, is not guaranteed or endorsed by the publisher.
