# Supplementary material for: Seasonal variation in the response of a monoecious crop to increased temperature and fertilizers
Source: Front Plant Sci. 2022 Oct 7;13:1012859. doi: 10.3389/fpls.2022.1012859 (PMC9585307; doi:10.3389/fpls.2022.1012859)
Supplement: Supplementary file 1 [file DataSheet_1.pdf]

Supplementary Material

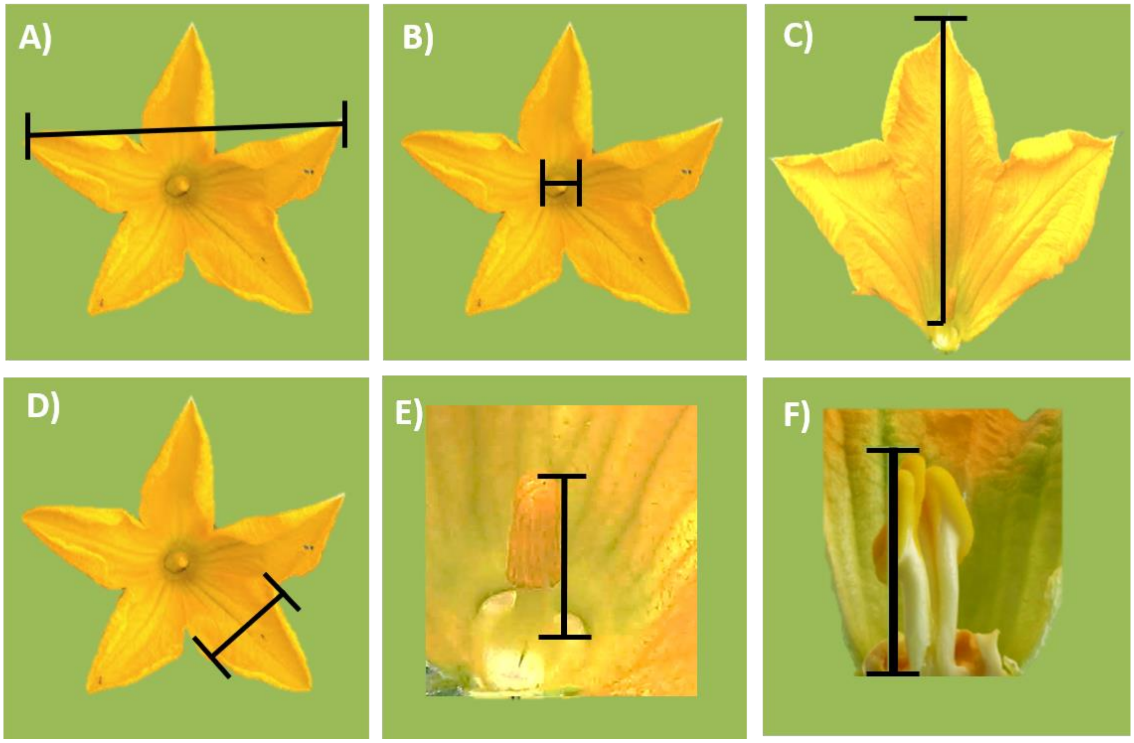

Supplementary Figure 1. Floral traits measured in pistillate and staminate flowers of *Cucurbita pepo*. **(A)** corolla diameter, **(B)** floral tube diameter, **(C)** floral tube length, **(D)** petal width, **(E)** length of stamen, **(F)** length of style-stigma.

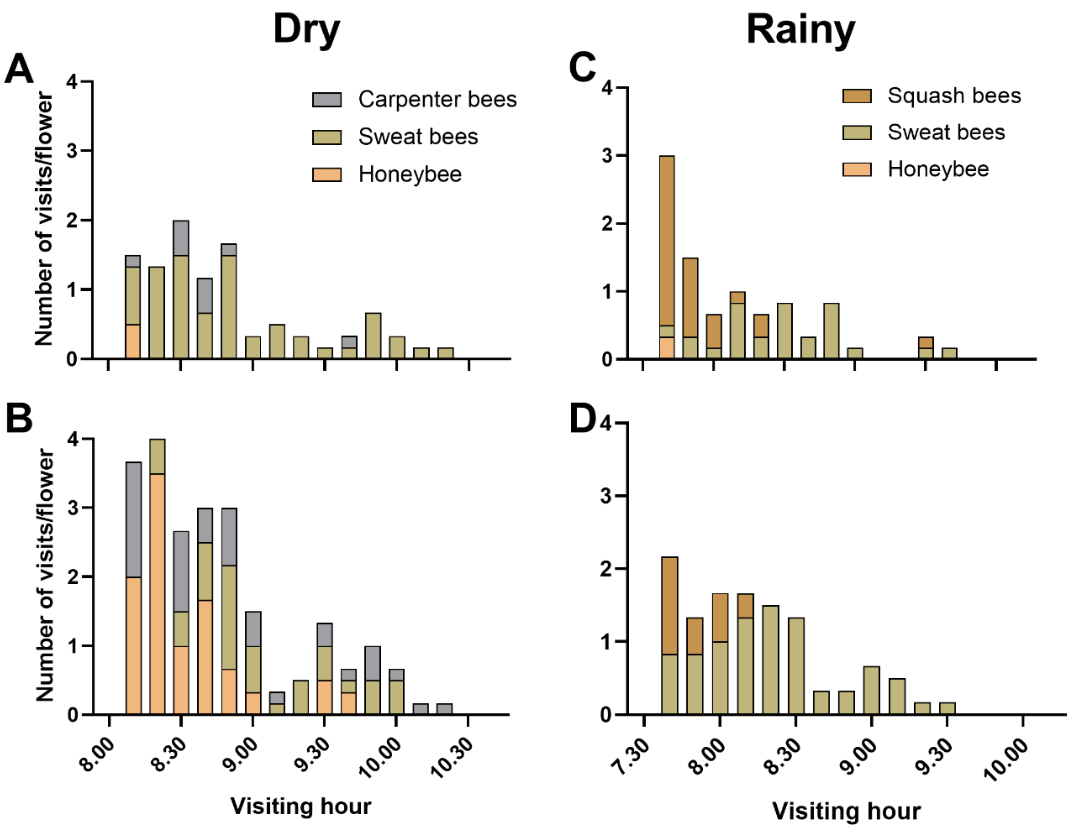

Supplementary Figure 2. Mean number of visits per flower in *C. pepo* flowers performed by different types of bees at 10-minute intervals from sunrise until the wilt of the flowers. Pistillate (A and C) and staminate (B and D) flowers during the dry (left) and the rainy (right) season. n=6 flowers.

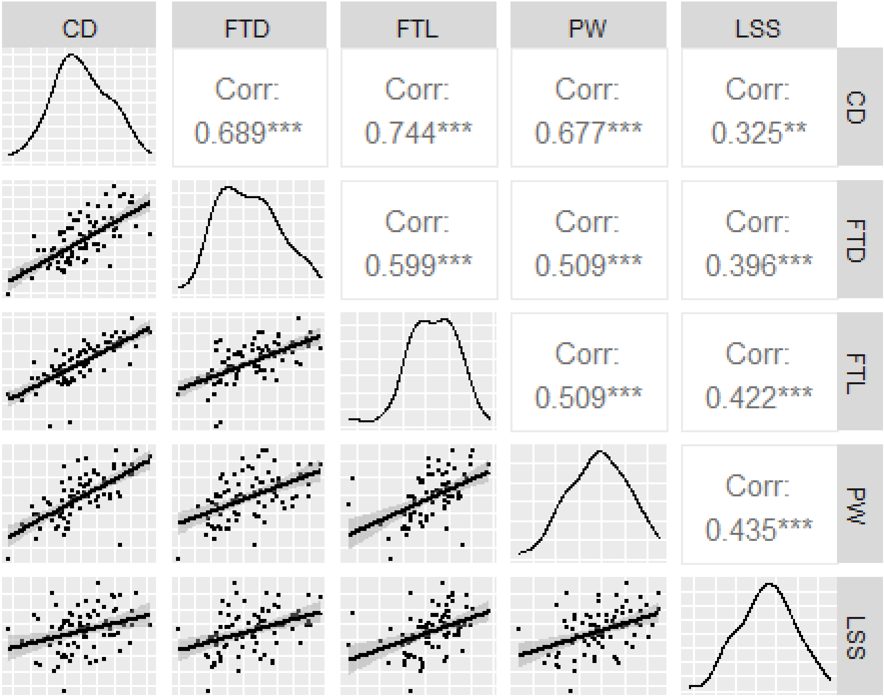

Supplementary Figure 3. Pearson correlations between all floral traits measured in pistillate flowers of *C. pepo* during the dry season. CD = corolla diameter; FTD = floral tube diameter; FTL = floral tube length; PW = petal width; LSS = length of style-stigma. The numeric values within the boxes are correlation coefficients between the paired traits. Statistical significance is indicated by asterisks, with *$p < 0.05$, **$p < 0.01$, ***$p < 0.001$. The straight lines in the lower diagonal are the best-fitted regression line, while the grey area indicates the 95% confidence intervals. Black curves in the mean diagonal represents the densities of the variables showing the data distribution.

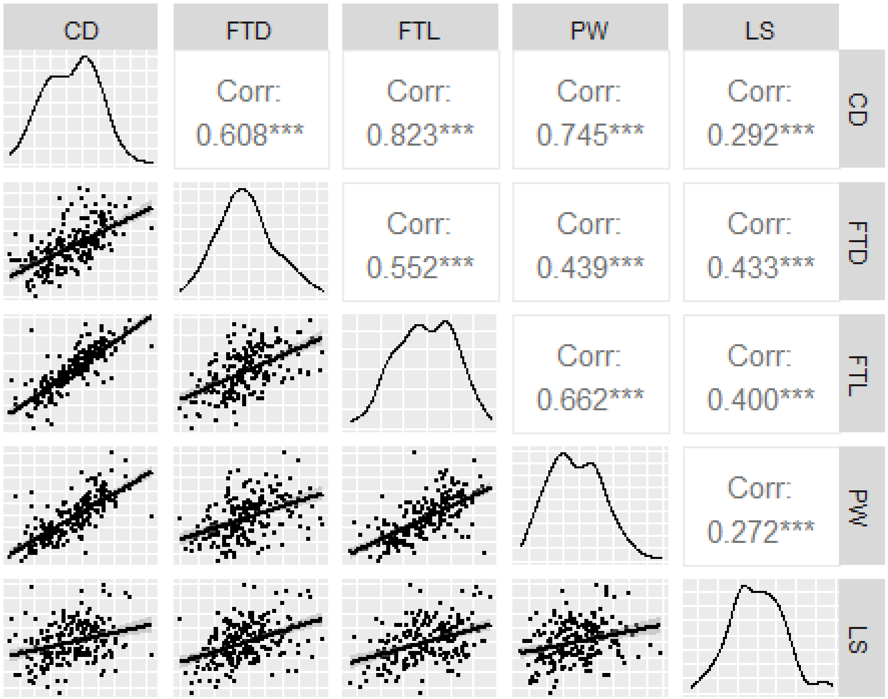

Supplementary Figure 4. Pearson correlations between all floral traits measured in staminate flowers of *C. pepo* during the dry season. CD = corolla diameter; FTD = floral tube diameter; FTL = floral tube length; PW = petal width; LS = length of stamen. The numeric values within the boxes are correlation coefficients between the paired traits. Statistical significance is indicated by asterisks, with *p < 0.05, **p < 0.01, ***p < 0.001. The straight lines in the lower diagonal are the best-fitted regression line, while the grey area indicates the 95% confidence intervals. Black curves in the mean diagonal represents the densities of the variables showing the data distribution.

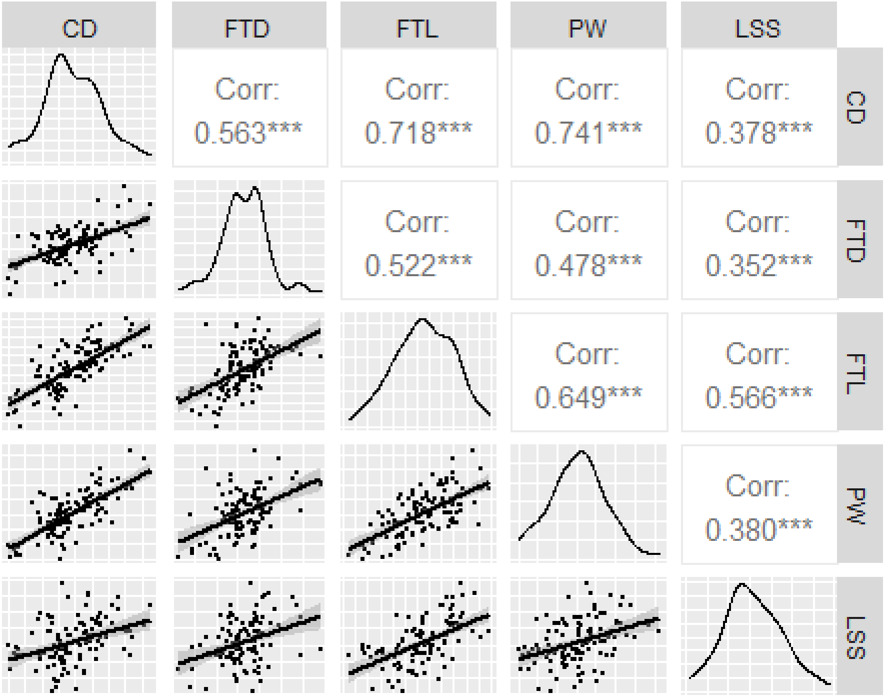

Supplementary Figure 5. Pearson correlations between all floral traits measured in pistillate flowers of *C. pepo* during the rainy season. CD = corolla diameter; FTD = floral tube diameter; FTL = floral tube length; PW = petal width; LSS = length of style-stigma. The numeric values within the boxes are correlation coefficients between the paired traits. Statistical significance is indicated by asterisks, with *p < 0.05, **p < 0.01, ***p < 0.001. The straight lines in the lower diagonal are the best-fitted regression line, while the grey area indicates the 95% confidence intervals. Black curves in the mean diagonal represents the densities of the variables showing the data distribution.

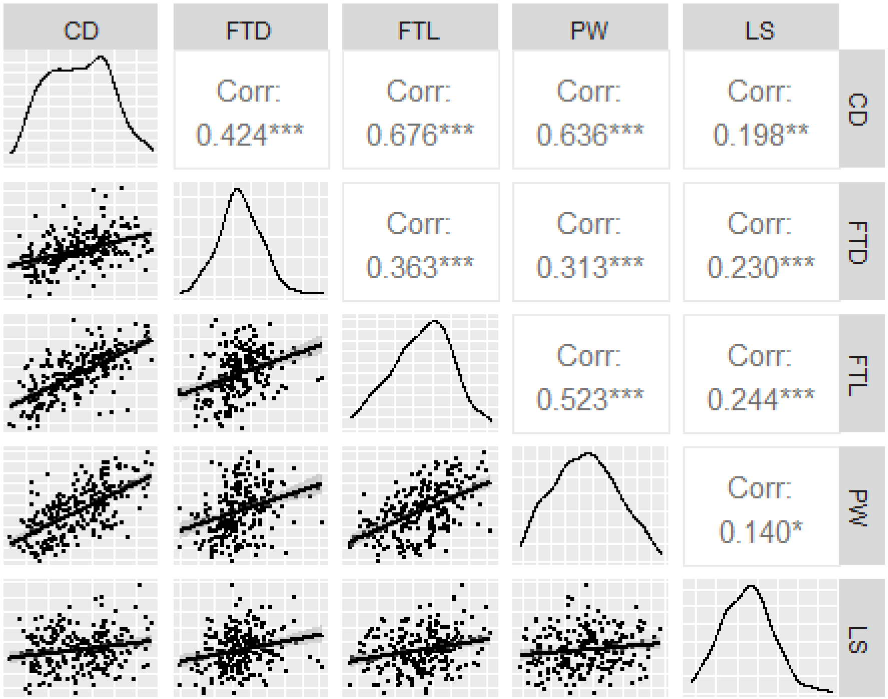

Supplementary Figure 6. Pearson correlations between all floral traits measured in staminate flowers of *C. pepo* during the rainy season. CD = corolla diameter; FTD = floral tube diameter; FTL= floral tube length; PW = petal width; LS = length of stamen. The numeric values within the boxes are correlation coefficients between the paired traits. Statistical significance is indicated by asterisks, with *p < 0.05, **p < 0.01, ***p < 0.001. The straight lines in the lower diagonal are the best-fitted regression line, while the grey area indicates the 95% confidence intervals. Black curves in the mean diagonal represents the densities of the variables showing the data distribution.

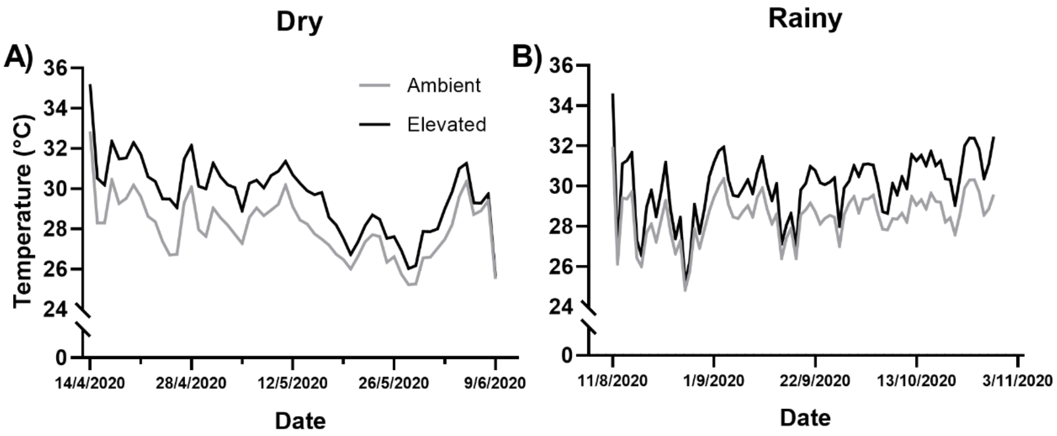

Supplementary Figure 7. Daily average temperature recorded inside (elevated temperature; black line) and outside (ambient temperature; gray line) the OTC during the dry **(A)** and the rainy season **(B)**.

frontiers

Supplementary Table 1. Effects of increased temperature (T) and fertilizer on vegetative and floral traits, legitimate floral visitors, and reproductive success of *C. pepo* plants during the dry season (Mean ± SD).

	No supplementary fertilizer		Organic fertilizer		Synthetic fertilizer	
**Parameters**	**Ambient**	**Elevated**	**Ambient**	**Elevated**	**Ambient**	**Elevated**
**Vegetative traits**						
Plant height	27.50 ± 2.12	34.70 ± 2.54	28.80 ± 3.08	34.40 ± 3.10	30.10 ± 2.51	34.40 ± 2.17
Number of leaves	12.00 ± 1.83	15.00 ± 2.36	14.60 ± 4.14	17.20 ± 4.83	18.20 ± 3.46	18.90 ± 3.38
Leaf area	226.65 ± 91.07	197.86 ± 41.87	220.02 ± 59.30	272.00 ± 52.39	337.77 ± 93.19	374.64 ± 120.08
**Floral traits**						
Pistillate flowers						
Timing of flowering	34.93 ± 6.86	34.82 ± 5.20	31.58 ± 4.60	32.13 ± 4.52	33.00 ± 3.46	33.35 ± 3.14
Number of flowers	2.00 ± 1.93	2.47 ± 1.18	2.07 ± 1.38	2.47 ± 1.41	2.87 ± 1.51	4.59 ± 2.48
Corolla diameter	71.12 ± 17.17	64.97 ± 11.56	65.10 ± 7.53	69.35 ± 15.52	73.59 ± 13.14	71.63 ± 19.32
Nectar volume	42.24 ± 20.41	53.35 ± 19.99	43.16 ± 12.30	48.90 ± 25.58	36.51 ± 28.35	51.39 ± 15.73
Sugar concentration	32.38 ± 4.73	34.95 ± 2.58	32.93 ± 4.60	31.84 ± 4.79	27.43 ± 4.53	39.30 ± 3.88
Flower sex ratio	0.19 ± 0.16	0.14 ± 0.09	0.08 ± 0.06	0.13 ± 0.07	0.15 ± 0.09	0.25 ± 0.12
Staminate flowers						
Timing of flowering	32.75 ± 6.90	29.71 ± 1.83	33.21 ± 6.27	29.47 ± 1.96	37.60 ± 7.26	29.82 ± 2.35
Number of flowers	9.38 ± 4.54	17.41 ± 5.59	25.50 ± 15.32	20.93 ± 10.39	19.93 ± 11.71	16.47 ± 10.62
Corolla diameter	70.35 ± 14.96	90.83 ± 17.96	63.81 ± 17.40	92.11 ± 16.68	68.52 ± 12.77	78.53 ± 14.09
Nectar volume	29.82 ± 11.56	22.11 ± 9.88	18.56 ± 4.25	33.88 ± 18.75	16.45 ± 6.74	27.09 ± 17.16
Sugar concentration	35.14 ± 2.42	37.36 ± 2.90	31.29 ± 3.80	32.07 ± 4.66	30.92 ± 6.08	38.03 ± 2.41
**Legitimate floral visitors**						
Pistillate flowers						
Number of legitimate visits	0.25 ± 0.50	1.67 ± 1.15	10.50 ± 2.52	4.25 ± 3.86	1.25 ± 0.96	2.00 ± 1.00
Duration of visit	5.00	12.60 ± 14.99	16.24 ± 15.28	14.35 ± 11.51	6.00 ± 1.22	8.20 ± 10.83
Staminate flowers						
Number of legitimate visits	8.38 ± 9.87	8.75 ± 6.67	4.78 ± 4.49	10.50 ± 9.14	8.17 ± 7.83	4.38 ± 4.27
Duration of visit	24.22 ± 88.89	22.16 ± 29.15	27.00 ± 51.04	18.44 ± 28.83	7.39 ± 7.95	22.40 ± 43.22
**Reproductive success**						
Fruit set	0.45 ± 0.52	0.35 ± 0.49	0.80 ± 0.45	0.58 ± 0.51	0.33 ± 0.49	0.16 ± 0.37
Number of seeds	165.20 ± 82.77	118.17±104.42	130.00 ± 73.54	113.00±106.07	147.40 ± 58.24	216.33 ± 65.29

Supplementary Table 2. Effects of increased temperature (T) and fertilizer on vegetative and floral traits, legitimate floral visitors, and reproductive success of *C. pepo* plants during the rainy season (Mean ± SD).

	**No supplementary fertilizer**		**Organic fertilizer**		**Synthetic fertilizer**	
**Parameters**	**Ambient**	**Elevated**	**Ambient**	**Elevated**	**Ambient**	**Elevated**
**Vegetative traits**						
Plant height	22.94 ± 3.33	24.97 ± 3.20	25.91 ± 3.28	27.06 ± 3.14	28.47 ± 5.88	32.19 ± 5.08
Number of leaves	10.76 ± 2.99	11.35 ± 2.15	13.18 ± 2.46	12.00 ± 1.97	14.22 ± 3.51	14.50 ± 4.25
Leaf area	57.42 ± 17.27	67.23 ± 17.74	101.37 ± 24.20	92.93 ± 28.22	221.27 ± 86.71	278.46 ± 52.47
**Floral traits**						
Pistillate flowers						
Timing of flowering	40.60 ± 7.97	40.06 ± 5.40	37.44 ± 7.88	40.29 ± 6.78	39.94 ± 4.92	37.22 ± 4.98
Number of flowers	2.00 ± 1.66	2.88 ± 1.22	1.76 ± 1.52	2.82 ± 1.47	3.39 ± 1.82	4.28 ± 2.72
Corolla diameter	71.40 ± 11.80	87.58 ± 12.99	79.87 ± 10.21	82.97 ± 15.15	94.55 ± 14.01	101.26 ± 15.44
Nectar volume	50.60 ± 36.88	62.18 ± 21.30	56.40 ± 15.31	49.09 ± 23.61	97.57 ± 53.38	60.31 ± 34.24
Sugar concentration	34.23 ± 3.78	32.74 ± 3.66	36.68 ± 2.89	32.88 ± 5.27	34.23 ± 4.09	31.67 ± 5.90
Flower sex ratio	0.17 ± 0.14	0.19 ± 0.09	0.14 ± 0.09	0.16 ± 0.08	0.24 ± 0.11	0.23 ± 0.09
Staminate flowers						
Timing of flowering	29.59 ± 4.18	28.59 ± 4.32	28.24 ± 3.31	31.65 ± 5.22	30.78 ± 3.73	31.50 ± 5.19
Number of flowers	10.35 ± 4.33	13.29 ± 4.66	11.82 ± 6.99	14.76 ± 3.58	12.22 ± 6.34	14.61 ± 7.35
Corolla diameter	66.93 ± 12.47	78.90 ± 8.95	70.73 ± 9.81	75.80 ± 9.68	70.06 ± 11.92	78.73 ± 10.06
Nectar volume	18.24 ± 7.15	18.61 ± 11.28	25.79 ± 18.77	16.61 ± 7.27	17.90 ± 9.59	15.42 ± 5.53
Sugar concentration	30.95 ± 5.16	32.96 ± 5.75	30.36 ± 5.28	32.54 ± 4.19	30.48 ± 5.81	33.72 ± 6.60
**Legitimate floral visitors**						
Pistillate flowers						
Number of legitimate visits	7.20 ± 4.44	11.33 ± 13.98	5.00 ± 5.18	7.00 ± 9.59	2.80 ± 3.03	8.83 ± 3.13
Duration of visit	34.55 ± 57.81	23.81 ± 50.92	18.73 ± 21.21	12.39 ± 14.52	14.86 ± 11.91	26.25 ± 29.52
Staminate flowers						
Number of legitimate visits	5.25 ± 3.74	7.60 ± 8.72	5.00 ± 4.15	5.67 ± 4.50	8.70 ± 7.21	3.50 ± 1.07
Duration of visit	73.24 ± 224.57	36.04 ± 112.49	79.71 ± 291.33	49.22 ± 102.26	58.55 ± 220.48	49.46 ± 51.75
**Reproductive success**						
Fruit set	0.14 ± 0.38	0.35 ± 0.49	0.33 ± 0.49	0.17 ± 0.38	0.47 ± 0.51	0.35 ± 0.48
Number of seeds	68.00	142.83 ± 69.82	101.60 ± 24.89	142.25 ± 14.38	186.21 ± 84.88	196.45 ± 74.82

Supplementary Table 3. Results of analyses testing the effects of temperature, fertilization, and their interaction on vegetative and floral traits, legitimate floral visitors, and reproductive success of *C. pepo* during the dry and the rainy seasons. ANOVA (F) or GLM (χ2) and p values are shown. Statistical significance is indicated by asterisks, with *p < 0.05, **p < 0.01, ***p < 0.001.

Parameters	Dry season			Rainy season		
	**Temperature**	**Fertilizer**	**T*F**	**Temperature**	**Fertilizer**	**T*F**
**Vegetative traits**						
Plant height	$F_{1,54}$=71.15***	$F_{2,54}$=0.97	$F_{2,54}$=1.54	χ^2_1=8.04**	χ^2_2=21.53***	χ^2_2=0.61
Number of leaves	χ^2_1=4.69*	χ^2_2=8.18 ***	χ^2_2=0.73	χ^2_1=0.02	χ^2_2=7.52***	χ^2_2=0.61
Leaf area	χ^2_1=0.65	χ^2_2=17.38 ***	χ^2_2=1.92	χ^2_1=1.81	χ^2_2=120.37***	χ^2_2=1.66
**Floral traits**						
Pistillate flowers						
Timing of flowering	χ^2_1=0.05	χ^2_2=1.97	χ^2_2=0.03	χ^2_1=0.01	χ^2_2=0.76	χ^2_2=1.74
Number of flowers	χ^2_1=4.80*	χ^2_2=7.13**	χ^2_2=0.59	χ^2_1=8.56**	χ^2_2=9.00***	χ^2_2=0.35
Corolla diameter	$F_{1,49}$=0.09	$F_{2,49}$=0.67	$F_{2,49}$=0.49	$F_{1,65}$=7.18**	$F_{2,65}$=13.39***	$F_{2,79}$=1.37
Nectar volume	$F_{1,26}$=1.95	$F_{2,26}$=0.09	$F_{2,26}$=0.12	χ^2_1=0.53	χ^2_2=1.45	χ^2_2=1.07
Sugar concentration	$F_{1,26}$=8.80**	$F_{2,26}$=0.25	$F_{2,26}$=7.097**	χ^2_1=2.56	χ^2_2=0.42	χ^2_2=0.16
Flower sex ratio	χ^2_1=3.92*	χ^2_2=13.71**	χ^2_2=4.26	χ^2_1=1.89	χ^2_2=13.85***	χ^2_2=0.76
Staminate flowers						
Timing of flowering	χ^2_1=16.73***	χ^2_2=1.67	χ^2_2=1.35	χ^2_1=0.92	χ^2_2=1.25	χ^2_2=1.41
Number of flowers	χ^2_1=2.32	χ^2_2=43.87***	χ^2_2=24.96***	χ^2_1=15.45***	χ^2_2=2.17	χ^2_2=0.14
Corolla diameter	$F_{1,73}$=30.22***	$F_{2,73}$=1.33	$F_{2,73}$=2.28	$F_{1,79}$=13.37***	$F_{2,79}$=0.15	$F_{2,79}$=0.71
Nectar volume	$F_{1,30}$=2.16	$F_{2,30}$=0.47	$F_{2,30}$=3.10	χ^2_1=1.77	χ^2_2=0.80	χ^2_2=0.93
Sugar concentration	$F_{1,30}$=7.06*	$F_{2,30}$=4.52*	$F_{2,30}$=2.15	χ^2_1=2.76	χ^2_2=0.07	χ^2_2=0.06
**Legitimate floral visitors**						
Pistillate flowers						
Number of legitimate visits	χ^2_1=1.30	χ^2_2=16.02***	χ^2_2=4.67*	χ^2_1=20.44***	χ^2_2=6.71**	χ^2_2=2.54
Duration of visit	χ^2_1=0.84	χ^2_2=3.46*	χ^2_2=0.66	χ^2_1=0.21	χ^2_2=6.89**	χ^2_2=3.33*
Staminate flowers						
Number of legitimate visits	χ^2_1=0.37	χ^2_2=3.41*	χ^2_2=11.44***	χ^2_1=1.46	χ^2_2=0.96	χ^2_2=11.25***
Duration of visit	χ^2_1=2.05	χ^2_2=5.84**	χ^2_2=8.14***	χ^2_1=7.45**	χ^2_2=0.54	χ^2_2=0.93
**Reproductive success**						
Fruit set	χ^2_1= 0.15	χ^2_2= 3.71*	χ^2_2=2.07	χ^2_1= 0.02	χ^2_2= 2.18	χ^2_2=1.15
Number of seeds	χ^2_1=0.61	χ^2_2=35.69***	χ^2_2=47.91***	χ^2_1=63.35***	χ^2_2=141.96***	χ^2_2=21.94***

Supplementary Table 4. Importance of legitimate floral visitors of *C. pepo* during the dry season.

Family	Species	Common name	Total of visits	Legitim visits to pistillate flowers	Legitim visits to staminate flowers	Importance value (%)
Apidae	*Peponapis* sp 1	Squash bee	0	0	0	0.0
	Peponapis sp 2	Squash bee	0	0	0	0.0
	Apis mellifera	Honeybee	258	1	178	1.8
	Xylocopa sp 1	Carpenter bee	87	10	77	23.1
	Trigona fulviventris	Stingless bee	1	0	0	0.0
Halictidae	*Halictidae* sp1	Sweat bee	155	65	68	74.4
	Halictidae sp2	Sweat bee	4	0	4	0.0
Vespidae	*Polybia occidentalis*	Social wasp	314	8	11	0.7
**Total**	**6 species**		**819**	**84**	**338**	**100.0**

Supplementary Table 5. Importance of legitimate floral visitors of *C. pepo* during the rainy season.

Family	Species	Common name	Total of visits	Legitim visits to pistillate flowers	Legitim visits to staminate flowers	Importance value (%)
Apidae	*Peponapis* sp 1	Squash bee	337	149	188	60.2
	Peponapis sp 2	Squash bee	92	37	55	16.0
	Apis mellifera	Honeybee	48	30	14	6.3
	Xylocopa sp 1	Carpenter bee	0	0	0	0.0
	Trigona fulviventris	Stingless bee	4	0	0	0.0
Halictidae	*Halictidae* sp1	Sweat bee	45	14	25	5.6
	Halictidae sp2	Sweat bee	113	27	68	11.8
Vespidae	*Polybia occidentalis*	Social wasp	15	0	0	0.0
**Total**	**7 species**		**654**	**257**	**350**	**100.0**
